# Quinine as a Hypnotic

**Published:** 1913-10-18

**Authors:** 


### THE EDITOR'S LETTER-BOX.

### Quinine as a Hypnotic.

*To the Editor of* The Hospital.

Sir,—I was somewhat interested in the letter from "F. A. M." which was printed in your issue of September 27 concerning the hypnotic qualities of quinine. I cannot, I fear, contribute much to the discussion of the question beyond the statement that quinine has certainly no hypnotic effect upon me, but, if anything, the opposite. But, since the experience of your correspondent is so very definitely stated, it may be of use to direct attention to the properties of quinine as determined by pharmacologists. In Dr. Hale White's "Materia Medica and Pharmacology," which is admittedly the standard (small) text-book on this subject, seventeen hypnotics and nine narcotics are enumerated; but quinine does not appear in either list. It does appear, however, seventeenth in a list of nineteen general cerebral stimulants; and it is, of course, well known that some drugs which in small doses stimulate the brain (*e.g.* alcohol, opium, cocaine), in large doses depress it. This, however, is not usually regarded as holding good for quinine, the symptoms of which in over-dosage form a very definite clinical picture known as cinchonism. Ringing in the ears, fulness in the head, deafness, and giddiness, passing on, in graver cases, to disturbance of vision, intense headache, delirium, coma, and failure of the heart or respiration; these are the results of ingesting poisonous doses of quinine, and, be it noted, that individual susceptibility to this drug is exceedingly marked, so that a dose which hardly affects one individual may make another one seriously ill.

With regard to the experimental pharmacology of quinine, Dr. Hale White cautiously remarks that small doses *are believed* (italics are mine) to stimulate the cerebrum; but that the results of experiments are so discordant as to be at present valueless. It does, therefore, appear as if "F. A. M.'s" experience must be exceptional; but that is no reason for discarding it without a fair trial. As far as clinical experience goes, a test of this remedy for insomnia as prescribed by "F. A. M." will certainly be made at the earliest opportunity by Yours faithfully,

General Practitioner.

